# An unusual failure of cardiac resynchronization therapy

**DOI:** 10.1007/s10840-022-01247-4

**Published:** 2022-05-16

**Authors:** Satoshi Higuchi, Henry H. Hsia

**Affiliations:** Division of Cardiology, Cardia Arrhythmia Service, University of California, San Francisco, 500 Parnassus Ave, MUE-434, San Francisco, CA 94143 USA

A 69-year-old man with non-ischemic cardiomyopathy and permanent atrial fibrillation underwent a pacemaker upgrade to a cardiac resynchronization therapy (CRT) defibrillator several years earlier. He experienced multiple inappropriate ICD shocks due to lead fracture and underwent a lead extraction with insertion of a new ICD lead performed at an outside hospital. Following the lead revision, the patient developed worsening heart failure, as well as intermittent pocket stimulation. An echocardiogram demonstrated a left ventricular (LV) ejection fraction of 35%, which was markedly reduced from the prior echocardiogram. He presented 1 year later for an elective defibrillator generator replacement due to battery depletion. His chest X-ray showed a rightward and inferior displacement of the ICD lead as compared to the pacemaker leads (panel A in Fig. 1). A CT angiogram demonstrated that the ICD lead was not in either the arterial or venous vasculature and had traversed under the first rib, puncturing directly into the ascending aortic arch (panel B in Fig. 1). The cardiothoracic surgery team was consulted for extraction of the LV ICD lead. However, the patient declined the surgery because of the high perioperative risk involving aortic root repair and potential aortic valve replacement. The patient was discharged home on chronic anticoagulation with serial echocardiographic follow-ups. Biventricular pacing and resynchronization therapy was re-established using the original LV epicardial lead as well as the old right ventricular endocardial pacemaker lead. He noted an immediate and marked improvement in his heart failure symptoms, which continued to improve over the subsequent months. A follow-up echocardiogram 5 months later showed a recovered LV ejection fraction of 50%.

**Fig. 1:**
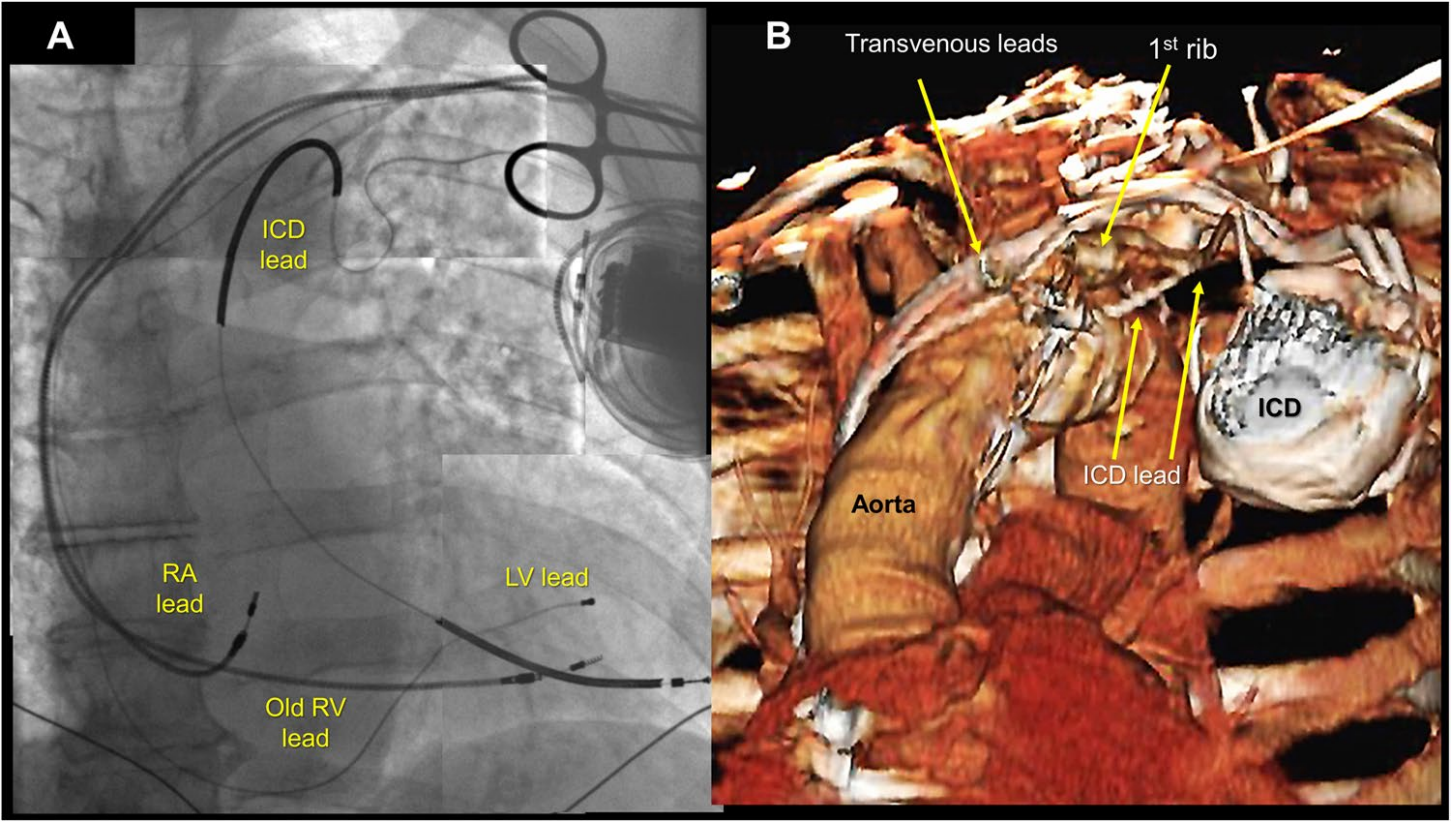
**A** Rightward and inferior displacement of the ICD lead. **B** ICD lead traversing under the first rib

This case illustrated a spectacular complication of a trans-arterial placement of the ICD lead into the aortic arch and a unique cause of CRT failure, with epicardial and endocardial pacing across the lateral LV wall. Although inadvertent arterial puncture can occur during routine access using the subclavian or proximal axillary vein [1], an LV lead placement can be prevented with a high index of suspicion, marked by an abnormal course of the wire on radiography after accessing the vasculature. A long wire should be advanced to the level of the inferior vena cava, below the right diaphragm before insertion of the sheaths and leads. Moreover, in cases with a challenging access, alternative approaches, such as an ultrasound-guided puncture or cut down of the cephalic vein would be of benefit to obtain access without risk of inadvertent arterial puncture.

## References

[1] van Gelder B, Bracke F, Oto A (2000). Diagnosis and management of inadvertently placed pacing and ICD leads in the left ventricle; a multicenter experience and review of the literature. Pacing Clin Electrophysiol.

